# *STUB1* mutations in autosomal recessive ataxias – evidence for mutation-specific clinical heterogeneity

**DOI:** 10.1186/s13023-014-0146-0

**Published:** 2014-09-26

**Authors:** Ketil Heimdal, Monica Sanchez-Guixé, Ingvild Aukrust, Jens Bollerslev, Ove Bruland, Greg Eigner Jablonski, Anne Kjersti Erichsen, Einar Gude, Jeanette A Koht, Sigrid Erdal, Torunn Fiskerstrand, Bjørn Ivar Haukanes, Helge Boman, Lise Bjørkhaug, Chantal ME Tallaksen, Per M Knappskog, Stefan Johansson

**Affiliations:** Department of medical genetics, Oslo University Hospital, Oslo, Norway; Center for Medical Genetics and Molecular Medicine, Haukeland University Hospital, Bergen, Norway; Department of Clinical Science, University of Bergen, Bergen, Norway; Section of Specialized Endocrinology, Medical Clinic B, Oslo University Hospital, Oslo, Norway; Faculty of Medicine, University of Oslo, Oslo, Norway; Department of Otorhinolaryngology, Head and Neck Surgery, Oslo University Hospital, Oslo, Norway; Department of Ophthalmology, Oslo University Hospital, Oslo, Norway; Department of Cardiology, Oslo University Hospital Rikshospitalet, Oslo, Norway; Department of Neurology, Vestre Viken Hospital, Drammen, Norway; KG Jebsen Center for Diabetes Research, Department of Clinical Science, University of Bergen, Bergen, Norway; Department of Neurology, Oslo University Hospital, Oslo, Norway; Faculty of Medicine, Oslo University, Oslo, Norway; K.G. Jebsen Centre for Neuropsychiatric Research, Department of Clinical Science, University of Bergen, Bergen, Norway

**Keywords:** *STUB1*, CHIP, HSC70, E3-ubiquitin ligase, ARCA, Ataxia, Hypogonadism

## Abstract

**Background:**

A subset of hereditary cerebellar ataxias is inherited as autosomal recessive traits (ARCAs). Classification of recessive ataxias due to phenotypic differences in the cerebellum and cerebellar structures is constantly evolving due to new identified disease genes. Recently, reports have linked mutations in genes involved in ubiquitination (*RNF216, OTUD4, STUB1*) to ARCA with hypogonadism.

**Methods and results:**

With a combination of homozygozity mapping and exome sequencing, we identified three mutations in *STUB1* in two families with ARCA and cognitive impairment; a homozygous missense variant (c.194A > G, p.Asn65Ser) that segregated in three affected siblings, and a missense change (c.82G > A, p.Glu28Lys) which was inherited in trans with a nonsense mutation (c.430A > T, p.Lys144Ter) in another patient. *STUB1* encodes CHIP (C-terminus of Heat shock protein 70 – Interacting Protein), a dual function protein with a role in ubiquitination as a co-chaperone with heat shock proteins, and as an E3 ligase. We show that the p.Asn65Ser substitution impairs CHIP’s ability to ubiquitinate HSC70 *in vitro*, despite being able to self-ubiquitinate. These results are consistent with previous studies highlighting this as a critical residue for the interaction between CHIP and its co-chaperones. Furthermore, we show that the levels of CHIP are strongly reduced *in vivo* in patients’ fibroblasts compared to controls.

**Conclusions:**

These results suggest that *STUB1* mutations might cause disease by impacting not only the E3 ligase function, but also its protein interaction properties and protein amount. Whether the clinical heterogeneity seen in *STUB1* ARCA can be related to the location of the mutations remains to be understood, but interestingly, all siblings with the p.Asn65Ser substitution showed a marked appearance of accelerated aging not previously described in *STUB1* related ARCA, none display hormonal aberrations/clinical hypogonadism while some affected family members had diabetes, alopecia, uveitis and ulcerative colitis, further refining the spectrum of *STUB1* related disease.

**Electronic supplementary material:**

The online version of this article (doi:10.1186/s13023-014-0146-0) contains supplementary material, which is available to authorized users.

## Background

Autosomal recessive hereditary cerebellar ataxias (ARCA) include a large number of rare degenerative disorders where gait disorder or clumsiness present as a key feature from an early age (characteristically before 20 years) [1]. Mutations in more than 20 genes have been found causal in these diseases. Despite the progress in gene identification, the molecular cause of disease still remains to be identified in about 40% of the families [1]. ARCAs are commonly classified according to mode of transmission and presence of additional features. In many cases, neurodegeneration with motor and cognitive deterioration are present in addition to ataxia.

Gordon Holmes syndrome (MIM 212840, hereditary cerebellar ataxia with hypogonadism) is one of these rare autosomal recessive syndromes combining ARCA with extracerebellar syndromes (hypogonadotrophic hypogonadism and often progressive dementia). Recently, Margolin et al. [2] identified mutations in the *RNF216* gene either alone or in combination with mutations in *OTUD4* as cause for this disease [2]. Interestingly, both genes encode enzymes in the ubiquitin pathway linking Gordon Holmes syndrome to disordered ubiquitation. Dysregulation of ubiquitination has also been linked to major neurodegenerative diseases such as Alzheimer and Parkinson [3,4]. These diseases have been associated with an accumulation of abnormal (misfolded) protein either as intracellular inclusions and/or in the extracellular space e.g. as amyloid depositis. The discovery of such excessive protein deposits, which in a normal state would be targeted to elimination by the cell defense (proteasome) system, has pointed to common mechanisms as cause for such general neurodegenerative diseases.

In 2013, Shi et al. reported, by exome sequencing, that mutations in the *STUB1* gene are a novel cause for Gordon Holmes syndrome [5]. The *STUB1* gene (STIP1 homology and U-box containing protein 1, E3 ubiquitin protein ligase) encodes CHIP, which is an E3 ubiquitin protein ligase. The role of ubiquitin ligases is to recognize the target protein to be ubiquitinated and mediate the attachment of ubiquitin. One affected sibling pair had a homozygous mutation predicted to lead to a missense change in the C-terminus of CHIP. The functional effect of the mutation was reported as reduced ubiquitin ligase activity. In another study, five additional *STUB1* mutations were reported in three different families [6]. All mutations were found to affect the ability of CHIP to promote N-methyl-D-aspartate receptor subunit degradation *in vitro*, which was suggested to be the underlying mechanism for the development of ARCA in these patients. Although all three Gordon Holmes associated genes (*RNF216, OTUD4*, *STUB1)* play a role in the ubiquitin system, the presence of dementia and white matter lesions on MRI has so far only been observed with *RNF216/OTUD4* mutations, illustrating some phenotypic diversity related to this syndrome [2]. Moreover, two groups recently reported additional families with ARCA due to *STUB1* mutations [7,8], further describing the heterogeneity of the syndrome.

**Figure 1:**
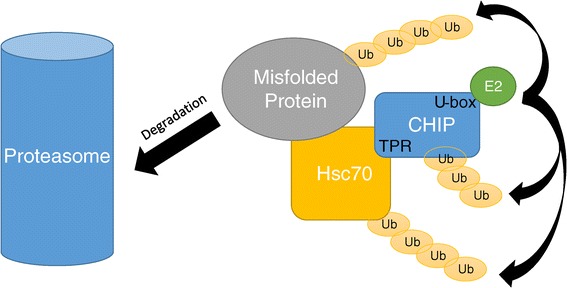
**The dual role of CHIP as both a co-chaperone and an E3 ligase targeting misfolded proteins to proteasome degradation.** CHIP binds to HSC70 by its TPR domain and bridges HSC70 to the misfolded protein. An E2 enzyme binds to the U-box domain and CHIP catalyses the ubiquitination reaction by attaching ubiquitin to the HSC70-client protein, targeting it to the proteasome. HSC70 and CHIP are also ubiquitinated, however this is not a signal for proteasomal degradation, but might play a role in their self-regulation.

CHIP is short for C-terminus of HSC70-interacting protein, thus it interacts with heat shock proteins (HSPs) that are highly conserved and abundantly expressed chaperone proteins with diverse functions. The most studied of these interacting proteins are HSC70, HSP70 and HSP90 [9]. CHIP functions both as a co-chaperone and an E3-ubiquitin ligase that couples protein folding and proteasome mediated degradation by interacting with heat shock proteins (e.g. HSC70) and ubiquitinating their misfolded client proteins thereby targeting them for proteasomal degradation (Figure 1).

CHIP itself comprises three functional domains: Tetratricopeptide repeat (TPR) domain, coiled-coil (CC) and U-box domain. The N-terminal TPR domain is the binding site for a wide range of proteins to be ubiquitinated by CHIP, including the HSPs (Figure 1). So far, more than 30 proteins have been identified as targets of CHIP [10]. The list includes ataxin-1, a protein that causes spinocerebellar ataxia type-1 (SCA1) when harboring an expansion of a polyglutamine tract [11]. CHIP has been found to be important for cellular differentiation and survival (apoptosis), and response to stress [10]. Further, studies in cell culture and post-mortem neurons have demonstrated a direct interaction between CHIP and ataxin-1, providing a link between CHIP and cerebellar ataxias [11]. Mouse models also support that CHIP may be important in preventing neurodegenerative diseases due to accumulation of abnormal proteins such as huntingtin or ataxin-3, and that haploinsufficiency of CHIP may accelerate such diseases [12,13]. Mice deficient in CHIP develop normally, but die prematurely with significant mortality observed in the peripartum and early postnatal periods. They demonstrate signs of specific behavioural impairments [10], and accelerated ageing, which is accompanied by signs of deranged protein quality control [14,15].

We investigated a consanguineous family with ARCA. Two affected brothers and their sister were found to share a homozygous missense variant in the tetratricopeptide domain of *STUB1* encoded CHIP. The variant was identified by homozygosity mapping using SNP-arrays followed by exome sequencing analysing genes in the homozygous region. To our knowledge, this is the third family with a mutation located in the TPR domain of CHIP. From our cohort of patients with ataxia, an additional patient with progressive ataxia and secondary infertility was selected for analysis, based on the phenotype similarities with the first family. Sanger sequencing demonstrated that this patient was compound heterozygous for a missense and a nonsense mutation in *STUB1*. The effect of the mutations on CHIP function was investigated by measuring CHIP ubiquitin ligase activity, using HSC70 as substrate for ubiquitination, as well as investigating effect of mutations on CHIP abundance in patient fibroblasts.

## Materials & methods

### Patients

Two affected brothers and their sister (Family 1) all presented with increasing gait disturbances and cognitive regression from 6 years of age, in addition to other non–neurological symptoms (Table 1). The parents are related (first cousins) of Arabic heritage originally from the Middle East, but living in Norway since the mid 1980’ies. The family is consanguineous and 7/8 grandparents descend from the same family. The affected siblings are presently 20–30 years old. Puberty/sexual developments have been un-remarkable, however menarche was somewhat delayed in the sister compared to other females in the family.

**Table 1:** **Clinical and radiological features of the four patients at examination date**

**Family-ID, Sex, Age at examination**	**P1[II-1], male, 26**	**P1[II-2], male, 30**	**P1[II-3], female, 20**	**P2, female, 45**
Substitution	N65S/N65S	N65S/N65S	N65S/N65S	E28K/K144*
Age of onset	2 years	CP diagnosis at birth	8 months	33 years
Onset symptom	Delayed development	na	Delayed development	Oligomennorhea, secondary infertility
Dysmorphic features at examination	Aged appearance	Aged appearance	Aged appearance	None
	Long slender fingers, increased space between digits four and five, adducted thumbs	Adducted thumbs	Minor unspecific facial dysmorphism	
			Long slender fingers, increased space between digits four and five	
First neurological symptom (age in years)	Gait impairment (17)	Gait impairment, dysarthria (12)	Gait impairment (15)	Gait ataxia, dysarthria (32)
Neurological signs & symptoms	Myokimies	Head tremor and generalized intermittent postural tremor	Dyspraxia	Cerebellar ataxia, Dysarthria, mild dysphagia
			Decreased tempo	
			Cerebellar ataxia, mild dysarthria	
	Cerebellar ataxia (17), dysarthria			
			Cognitive impairment	
			Epilepsy until 2 years of age	
		Cerebellar ataxia, dysarthria, dysphagia		
				Decreased tempo
		Increased muscle tone (rigidity and gegenhalten)		
	Dyspraxia			
		Distal muscle atrophy		
		Cognitive impairment		
	Increased muscle tone (rigidity)			
	Cognitive impairment			
Disability score*	5	5 (from 22 years)	2	4
MR findings (at examination)	Cerebellar hypoplasia, thin posterior corpus callosum, mild thinning of pons	Severe cerebellar atrophy, thin corpus callosum, thin pons	Cerebellar hypoplasia, thin pons and corpus callosum	Cerebellar hypoplasia, mild thinning of pons, “empty sella”
Ophthalmological findings	Horizontal nystagmus	Left sided chronic iridocyclitis with secundary glaucoma; Oculomotor dyspraxia with saccadic pursuit	Horizontal nystagmus; mild retinal atrophy	Results not available
Endocrinology	Increased anti TPO		Delayed menarche for family	Secondary infertility Hypothyroidism
	Diabetes type I			
				Diabetes type 2
Other	Alopecia	Ulcerative colitis	Slight presbyacusis	Pancreatitis
	Slight presbyacusis			

*Disability score → 0: no functional handicap; 1: no functional handicap but signs at examination; 2: mild, able to run, walking unlimited; 3: moderate, unable to run, limited walking without aid; 4: severe, walking with one stick; 5: walking with two sticks; 6: unable to walk, requiring wheelchair; 7: confined to bed.

A search in our ataxia database revealed one female patient with secondary infertility due to hypogonadotrophic hypogonadism, in addition to ataxia. She was included in the study due to phenotypic similarity with the patients described in the first publication by Shi et al. [5]. The patient originates from Sri Lanka. Her parents are unrelated but from the same geographical area. She was completely healthy until the age of 25, when she developed secondary infertility. The first signs of ataxia started at age 33.

Informed written consent was obtained from all participants. The study was approved by the Regional Committee for Medical and Research Ethics, South East Norway (ref. no. 2012/1425b), and adhered to the tenets of the Declaration of Helsinki.

### Genotyping and sequencing

Genome wide SNP genotyping was performed with the Genome Wide Human SNP array 6.0 (Affymetrix, Santa Clara). Whole genome homozygozity mapping was performed using PLINK v1.07 [16,17] searching for any region >2 Mb, with minimum of 30 SNPs and less than four heterozygous calls. Whole exome capture and paired-end 100 nt sequencing was performed at HudsonAlpha Institute for Biotechnology (Huntsville,AL) as described in (Haugarvoll 2013). The 8.7 Giga-bases of aligned sequence data resulted in 55X median coverage of the target capture regions, with more than 96% of target bases covered a minimum of 8X. PCR duplicates were removed with PICARD (http://broadinstitute.github.io/picard/) and the Genome analysis toolkit [18] was used for base quality recalibration and variant calling using a minimum threshold of 8X sequencing depth and quality score ≤ 30. Annovar [19] was used for variant annotation. Variant prioritization was performed as described in [20] based on an autosomal recessive model, filtering against variants identified in more than 100 Norwegian exome-resequencing samples (obtained using the same whole exome sequencing pipe-line) and variants present at >0.5% allele frequency in the 1000 Genomes database. Variants were verified by Sanger sequencing using the BigDye terminator kit and the ABI7900 Genetic Analyzer. For the proband in Family 2, all exons and intron/exon boundaries in *STUB1* were sequenced by Sanger sequencing (primers and conditions available upon request). To test whether the mutations found in Patient 2 were located on different strands we used the TOPO® TA Cloning® Kit (Invitrogen, Life technologies, 11329-H07E-25, California) to clone PCR-products spanning both mutations, followed by Sanger sequencing of the clones. *STUB1* reference sequence (RefSeq) used: NM_005861.2

### RNA-studies

Total RNA was purified from blood using the Tempus system (Life Technologies, California) or from cultured fibroblasts using the RNEasy-kit (Qiagen, Germany). Reverse transcription and cDNA synthesis were performed using the SuperScript^®^ VILO^™^ cDNA Synthesis Kit (Life Technologies, California). Expression of the *STUB1* gene was measured by qPCR using MGB-probes (Life Technologies, California) and gene expression was normalized using beta-actin and GADPH as endogenous controls. Relative expression was calculated using the delta Ct- method.

### Plasmids and constructs

The full length cDNA encoding CHIP from purchased vector pMXs.EXBi-STUB1-IRES-Puro (Cyagen Bioscience Inc., California) was cloned into bacterial expression vector pETM-41 (EMBL, Heidelberg, Germany), and the mammalian expression vector pcDNA3.1/V5-HisB (Invitrogen, California). Resulting constructs pETM-41-CHIP and pcDNA3.1V5-HisB-CHIP were used as templates for site directed mutagenesis (Quick change kit, Stratagene, California) generating plasmids containing the following CHIP point mutations (E28K), (N65S), (K144*) and (T246M). The authenticity of each construct was confirmed by DNA sequencing.

### Protein expression and purification

Hisx6-MBP-tagged CHIP, wild type and mutant recombinant protein, were expressed in BL21-CodonPlus (DE3)-RP Competent Cells (Agilent, California). Briefly, transformed cells were grown in LB medium added 0.2% glucose until A600 reached 0.6, and induced with 0.5 mM isopropyl-β- D-thiogalactopyranoside for 5 hours at 30°C. Cells were harvested and lysed by sonication. The Hisx6-MBP-tagged proteins were purified using Amylose resin (New England Biolabs, Massachussets), according to manufacturer’s instruction. For tag-free CHIP proteins, the Hisx6-MBP-tagged fusion proteins were cleaved by tobacco etch virus protease (TEV) for 2 hours at room temperature.

### Expression of CHIP proteins by the *in vitro* coupled transcription/translation system

CHIP-WT and CHIP-N65S were expressed *in vitro* in a coupled transcription/translation system (TNT T7 Quick-coupled Transcription/Translation system; Promega) using 2 μg plasmid DNA and in the presence of [^35^S]Met (10 μCi), 20 mM DTT and 40 μl of rabbit reticulocyte lysate. Expression was performed at 30°C for 90 min, and samples analyzed by SDS-PAGE and autoradiography.

### *In vitro* ubiquitination assay

*In vitro* ubiquitination reactions were set up as previously described [5]. Ubiquitination immediately followed after production of recombinant MBP-CHIP forms, and after cleaving 2 h at room temperature with 1 μg of TEV per 10 μg of protein, if tag-free CHIP was used in the analyses. In a total volume of 20 μl of 50 mM Tris HCl (pH 7.5), 0.6 mM DTT and 2.5 mM Mg-ATP (Sigma Aldrich, Missouri), 2.5 μM of recombinant CHIP was incubated with 50 nM Ube1 (Boston Biochem, E-305, Massachussets), 2.5 μM UbcH5c (Boston Biochem, E2-627, Massachussets), 0.7 μM HSC70 (SinoBiological Life technologies, 11329-H07E-25, California), and 250 μM ubiquitin (BostonBiochem, U-100H, Massachussets), for 1 h at 37°C. Samples were analyzed by SDS-PAGE (4-12%) and immunoblotting using anti-HSC70 (Enzo, ADI-SPA-815, New York) or anti-CHIP (LifeSpan Biosciences, LS-C137950, Washington) antibodies.

### Fibroblast culture

Four millimeter punch biopsies were obtained from the skin of the ventral aspect of the forearm of patients P2, P1 and the father of P1 (F-P1) in local anesthesia and shipped to the laboratory in transport medium. The skin biopsies were cultured and expanded in Amniochrome II Basal medium with Amniochrome II Modified Supplement (Lonza) at 37°C in 5% Co2. High confluent cells were washed with PBS and harvested in RIPA Buffer with 1X Halt Protease Inhibitor Cocktail, and analyzed by SDS-PAGE (10%) and immunoblotting using anti-CHIP and anti-actin (Santa Cruz Biotechnology, sc-1615, California) antibodies.

## Results

### Clinical features

All clinical features are summarized in Table 1.

Family 1: There is adult onset diabetes in several members on the paternal side, including the father, but no other instances of ataxia or mental impairment in the family. Several family members including patient P1[II-1] have thalassemia minor. All siblings were born after uneventful pregnancies except the youngest who was born prematurely.

The index case (Patient P1[II-1]) is a 26 year old male who was considered normal from birth until his grandmother remarked delayed development (motor and cognitive) when the boy was 2 years old. He started walking independently at age 2 ½. He has always had an unsteady gait with progression of ataxia particularly from the early teens. Motor function was reasonable until about 7 years of age (he could use a bicycle, play soccer and run, but slower than other children). Presently, he can walk independently for short distances, but prefers using a walker. The family moved to Norway when he was 6 years old and he has learnt a little Norwegian. Recently, he has had increasing difficulties with expressive language. He did not attend regular school and has never learned to read or write. He experienced normal pubertal development and physical appearance. External genitals are normal for an adult male. He developed diabetes type I from age 16. His hair was normal until 4–5 years of age after which he developed near total alopecia, which was treated with systemic steroids with little clinical effect. He has no dysmorphic features, but his physical appearance resembles that of a much older person than his chronological age of 26 years.

**Figure 2:**
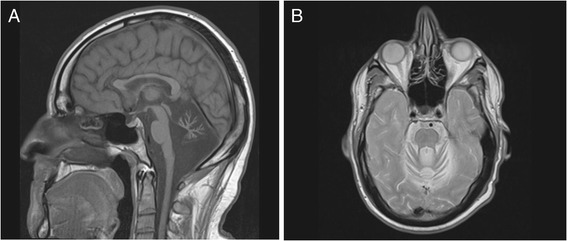
**Cerebral MRI (1.5 Tesla). (A)** Cerebral MRI (T1 serie, midline sagittal) of the proband in Family 1 at the time of investigation. Severe atrophy of the whole cerebellum and the anterior part of the corpus callosum. **(B)** Same examination, but T2 axial scan at the level of the superior cerebellar peduncle. There is an atrophy of both cerebellar hemispheres with widened sulci, and vermis atrophy. The fourth ventricle is moderately dilated. There are a few diffuse hyperintensity signals in the brainstem. The cerebral hemispheres look normal.

Endocrine investigations at age 26 shows normal pituitary (marginally raised prolactin), testosterone (SHBG at upper reference limit for laboratory), and adrenal function. Anti-TPO was increased to six times the upper limit of normal (ULN), however with clinical and biochemical normal thyroid function, and no goitre. Other endocrine autoantibodies were normal. Pure tone audiometry indicated very mild sensorineural hearing loss in the high frequencies from 4000 Hz, as seen in presbyacusis or after noise exposure. Cardiac examination including echocardiography, and bone mineral measurements were normal. Cerebral MRI showed severe cerebellar atrophy, atrophy of the corpus callosum particularly pronounced anteriorly, and a slight atrophy of the pons and brainstem (Figure 2A and B).

The elder brother (P1[II-2]), now 30 years of age, has a similar clinical picture as the index case, however, his neurological condition appears more severe. He is still ambulatory with a walking chair and communicates verbally. He was initially diagnosed with cerebral paresis in his native country. He developed therapy resistant ulcerative colitis at age 22 treated with proctocolectomy. Asymptomatic uveitis developed in his left eye 26 years old. There has been marked neurological progression with worsening ataxia and a decline in higher mental functions. The parents informed us that he had a normal pubertal development and he has the appearance of a normal adult male, though strikingly older looking than his biological age. He has refused further clinical and supplementary investigations at this point.

The younger sister (P1[II-3]), now 18 years of age, has a similar clinical picture albeit milder than her brothers. She was born prematurely and had epilepsy (generalized) 8 months old. She was medicated until 2 ½ years of age and has not had seizures since. She has ataxia and impaired cognition, but has learnt to write her name in school and is able to read a few words. The parents were certain she had the same condition as her brothers when she was eight months old. She walked independently at age 2 ½. Motor development has been slow. She developed cerebellar ataxia with increasing gait impairment from age 15, but is still ambulatory without walking aids, and has very moderate extremities’ ataxia. She can walk, but is unable to run. She speaks Arabic and some Norwegian, does not know how to add but can count.

Sexual development has been normal with menarche at age 15 followed by regular periods. Physical appearance is that of a normal female with very slight and unspecific dysmorphic features, however, with a much more aged appearance than expected for an 18 year old woman. Endocrine investigations at age 18 shows normal pituitary function, normal sex hormones and adrenal function. We found no indications of autoimmune endocrinopathies. Pure tone audiometry indicated very mild sensorineural hearing loss in the high frequencies comparable to that found in her brother. Cardiac examination including echocardiography and bone mineral measurements were normal.

Patient P2 is a 45 year old woman of Sri Lankan descent living in Norway. She is the youngest of three children of unrelated healthy parents and the only affected family member. She developed ataxia after the age of 30, but her primary symptoms presented as secondary infertility due to hypogonadotrophic hypogonadism. Development was normal during childhood and adolescence. She had normal sexual development with menarche 14 years old and childbirth 25 years old. After giving birth, she has had oligomenorrhea and secondary infertility. Investigations showed deficits in pituitary function and “empty sella” on MRI. Neurological symptoms started at about age 32 with increasing difficulties with walking and ataxia. The condition has been slowly progressive. She is still ambulatory, but needs the support of a walker due to impaired balance.

### Whole genome genotyping and exome sequencing identify a homozygous *STUB1* mutation segregating with ARCA in Family 1

The consanguineous structure of Family 1 suggested recessive inheritance and we therefore performed whole genome genotyping to search for regions of homozygosity in the three affected siblings and their parents. We identified two regions of homozygosity shared identity by descent among the three affected siblings: a 6.6 Mb area on chromosome 5 (25,455,664-32,08505, NCBI Build 36.3) and 2.7 Mb region on chromosome 16 (0–2,764,985). None of the areas contained known ARCA genes or other obvious candidate genes. We next performed whole exome sequencing in the index patient (Additional file 1: Table S1). This identified 20438 genetic variants of which 429 were non-synonymous and not found in 100 Norwegian exomes or in the 1000 Genomes database at > 0.5% allele frequency. Only one variant, c.194A > G in *STUB1* was located in a region shared identical by descent in all three siblings and heterozygous in each parent. Sanger sequencing confirmed that the mutation is homozygous in all three siblings and heterozygous in both parents. The c.194A > G mutation is located in *STUB1* exon 2 and is predicted to cause an Asparagine (N) to Serine (S) amino acid substitution (p.Asp65Ser, NM_005861) affecting a highly conserved residue. The mutation is predicted as deleterious by SIFT, Poly-Phen 2 and Mutation Taster and is not found in 1000G or dbSNP.

### Identification of compound heterozygous *STUB1* mutations in Family 2

Sanger sequencing of the proband in Family 2 identified two heterozygous, previously undescribed variants, in *STUB1*; c.82G > A and c.430A > T. The c.82G > A mutation is predicted to encode a Glycine to Lysine substitution at residue 28 (p.Glu28Lys) while the second mutation results in a premature stop codon, p.Lys144Ter in exon 3. The mutations were confirmed to be located on different strands by sequencing of clones derived from PCR-products spanning both mutations.

### Location of the CHIP mutations p.Asn65Ser (observed in Family 1), p.Glu28Lys, and the truncated form p.Lys144Ter (both observed in Family 2)

**Figure 3:**
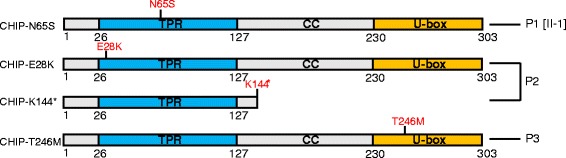
**Functional domains of the CHIP protein and illustration of amino acid substitutions/deletions found in patients.** Presentation of the CHIP E3-Ligase with its three functional domains: Tetratricopeptide repeat (TPR), coiled-coil (CC) and U-box. Patient 1 (P1[II-1]) is homozygous for a point mutation resulting in CHIP-N65S located in the TPR domain. Patient 2 (P2) is compound heterozygous for two point mutations; one resulting in CHIP-E28K in the TPR domain and another causing the deletion mutant CHIP-K144*, a truncated protein lacking most of the CC domain and the entire U-box domain. The mutation resulting in the CHIP-T246M mutant is located in the U-box domain and has previously been described [5] and indicated here in Patient 3 (P3).

The p.Asn65Ser (CHIP-N65S) and p.Glu28Lys (CHIP-E28K) mutations are located in the TPR domain important for chaperone interactions (Figure 3). The Asn-residue at position 65 is highly conserved (from human to *C. elegans*) and previously shown to be directly involved in binding of substrates such as Smad1, HSC70, HSP70 and HSP90 [21]. The non-synonymous heterozygous change in Family 2 alters a glutamic acid (E) to lysine (K) in position 28, close to a second critical residue for substrate binding (Lys30) in the TPR domain [21], and predicts a change from a negatively to a positively charged amino acid. The second mutation seen in patient 2 is predicted to lead to a premature stop codon (CHIP-K144*) and may result in loss of translation into a functional protein. Just recently, Shi and colleagues reported one family with another homozygous *STUB1* mutation as the cause of ataxia and hypogonadism in two siblings of a consanguineous marriage [5]. Interestingly, this mutation (p.Thr246Met) is located in the U-box domain (Figure 3) and is thus more likely to directly impair the ubiquitin ligase activity, while retaining normal substrate binding of CHIP. We decided to include this mutant in our functional studies for comparison.

### Decreased levels of steady-state CHIP observed in patient fibroblasts

**Figure 4:**
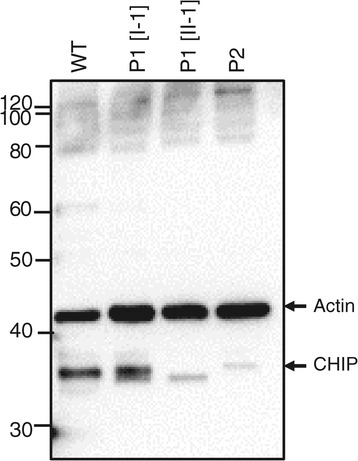
**Differential levels of CHIP protein in fibroblasts from patients.** Fibroblasts from Patient 1 (P1[II-1]) and Patient 2 (P2) show lower steady-state levels of CHIP protein compared with normal fibroblasts (WT), as analyzed by SDS-PAGE and immunoblotting using CHIP-specific antibody. In addition, the band corresponding to CHIP-N65S mutant in P1 reveals slightly faster migration rate on SDS-PAGE compared to the CHIP-WT band, probably due to protein conformational changes induced by the mutation. In fibroblasts from the father of Patient 1 (P1[I-1]), a heterozygous carrier of the N65S mutant allele, a double band can be observed (CHIP-WT and CHIP-N65S). In P2 a weak band most likely corresponding to CHIP-E28K is detected, while the band corresponding to the lower molecular weight CHIP-K144* form of ~16 kDa is not observed on this blot. Actin-specific protein bands are shown to compare the relative amounts of total protein loaded per lane.

**Figure 5:**
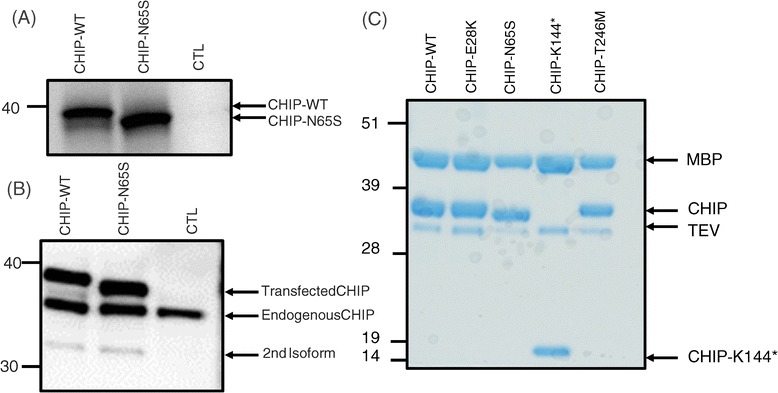
**CHIP-N65S causes a migration shift when analyzed by SDS-PAGE. (A)** CHIP-WT and CHIP-N65S were translated in a TNT coupled transcription/translation system in the presence of [^35^S]Met, as described in Material & Methods. Samples were analyzed by SDS-PAGE and autoradiography. A double band can be observed for CHIP-WT, in which the lower band migrates at the same rate as CHIP-N65S. CTL; empty vector as negative control. **(B)** CHIP expression of transfected HEK293 cells with CHIP-WT or CHIP-N65S, tagged with V5 and His, and detected by SDS-PAGE and immunoblotting using anti-CHIP. Endogenous CHIP is observed at 35 kDa. The shift is observed only for transfected CHIP. A secondary isoform of CHIP appears at 32 kDa, lacking the first 72 amino acids, only observed *in vitro* [9]. CTL; empty vector as negative control. **(C)** Recombinant WT and CHIP variants expressed and purified from *E. coli* as MBP-fusion proteins and cleaved by TEV protease as described in Materials & Methods. Samples were analyzed by SDS-PAGE and coomassie staining. The migration shift is only observed for CHIP-N65S. CHIP-K144* appears at a lower molecular weight (truncated version of 16 kDa).

Immunoblot analysis using a CHIP specific antibody shows that fibroblasts derived from Patients 1 (P1[II-1]) and 2 (P2) have much lower steady state levels of CHIP protein compared to both normal fibroblasts and the healthy father of Patient 1 (P1[I-1], Figure 4). In the compound heterozygous Patient 2, only a weak band corresponding to CHIP-E28K is detected. No lower molecular weight form corresponding to an estimated ~16 kDa CHIP-K144* truncated form could be detected, suggesting that CHIP-K144* is not present as a mature protein in the patient. This was later confirmed by quantitative RT-PCR (data not shown). For Patient 1 P1[II-1] the band corresponding to CHIP-N65S migrates slightly faster during SDS-PAGE compared to the CHIP-WT band (Figure 4). Similarly, in fibroblasts from the heterozygous carrier of the N65S mutant allele (P1[I-1]), a double band with different migration pattern can be observed (CHIP-WT and CHIP-N65S). This migration difference for CHIP-N65S is probably due to a protein conformational change induced by the mutation, as we observe the same slight migration difference for CHIP-N65S when it is i) expressed in an *in vitro* rabbit reticulocyte protein expression system, ii) expressed in HEK293 cells transfected with CHIP-N65S encoding plasmids, and also when iii) expressed in *E. coli* as recombinant CHIP-N65S (Figure 5). Since the migration difference is also observed for *E. coli* expressed and purified proteins, it is unlikely that the shift is caused by a post-translational modification, but rather due to conformational change induced by this particular amino acid substitution.

To investigate whether the mutations were expressed at the transcript level, we sequenced cDNA derived both from peripheral blood and cultured fibroblasts from Patient 1, his heterozygous father and Patient 2. The non-synonymous mutations were all detected, but no trace of the CHIP-K144* could be found. Quantitative RT-PCR showed normal levels (compared to WT) in both the heterozygous and homozygous carriers in Family 1, and approximately 50% lower level in the compound heterozygous Patient 1 (data not shown). These results suggest that the CHIP-K144* mutant allele is degraded at the transcript level, possibly due to nonsense mediated decay.

### CHIP-N65S demonstrates reduced ubiquitin ligase activity

**Figure 6:**
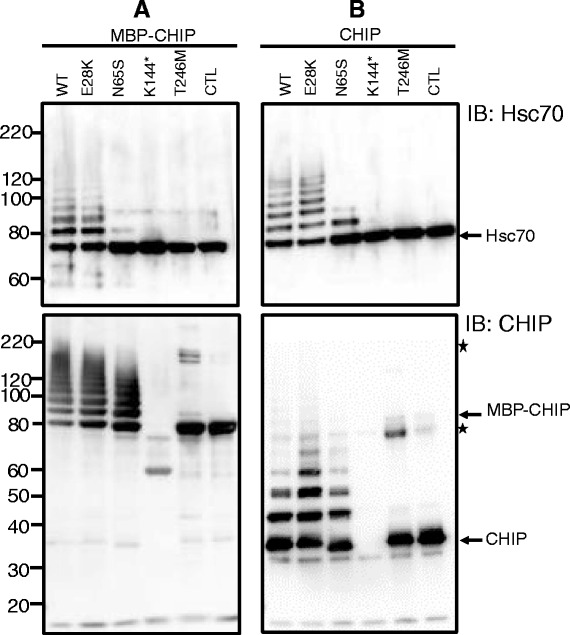
**Different E3 ubiquitin ligase activity is observed for various CHIP mutants.** *In vitro* ubiquitination was assessed using CHIP-WT and CHIP-mutant forms as E3 ligases and HSC70 recombinant protein as substrate for ubiquitination. Samples were analyzed by SDS-PAGE followed by immunoblotting using HSC70- and CHIP-specific antibodies. A reaction with WT-CHIP and without ubiquitin was used as a negative control (CTL). Both the levels of ubiquitination of HSC70 and auto-ubiquitination of CHIP itself was investigated using MBP-CHIP fusion protein **(A)** and tag-free (cleaved) CHIP **(B)**. The lower molecular weight CHIP-K144* deletion mutant were detected as a MBP fusion protein, but not as a tag-free mutant, presumably due to reduced protein stability after removal of the MBP. The *asterisks* indicate CHIP forms mostly observed for CHIP-T246M and possibly representing protein dimers.

To study whether the mutations affect substrate binding and ubiquitin ligase activity, we expressed and purified CHIP-WT, CHIP-E28K, CHIP-N65S, CHIP-K144* and CHIP-T246M, both as recombinant MBP-fusion proteins and tag-free (cleaved) CHIP. The *in vitro* ubiquitination activity of each mutant was assessed using HSC70 recombinant protein as substrate. As can be seen in Figure 6 (top panel), CHIP-K144* and CHIP-T246M fail to ubiquitinate HSC70 *in vitro*, while CHIP-E28K is able to ubiquitinate HSC70 at the same level as CHIP-WT. Interestingly, the ability of CHIP-N65S to ubiquitinate HSC70 appears significantly impaired (top panel). To investigate whether this was due to a defect in binding to HSC70, and not due to a defect in ubiquitin ligase activity, we also measured the intrinsic autoubiquitination ability of each of the CHIP mutants (Figure 6, lower panel). As previously described by others [5], CHIP-T246M has no ubiquitin ligase activity and showed no autoubiquitination. In contrast, both mutants with affected TPR domain (lower panel) showed levels of autoubiquitination indistinguishable from the WT protein and thus, appear to have intact ability to ubiquitinate. Therefore, low substrate affinity is the more likely mechanism for the reduced HSC70 ubiquitination observed for CHIP-N65S. The lower molecular weight K144* deletion mutant was detected as a MBP fusion protein, but not as a tag-free mutant, presumably due to reduced protein stability after removal of the MBP.

## Discussion

We used a combination of homozygosity mapping and exome sequencing to identify the disease causing DNA variant in a consanguineous family with cerebellar ataxia. During the course of our investigations, four research groups reported *STUB1* mutations as the disease cause in families with ARCA with/without hormonal aberrations and auxilliary clinical findings [5,6,8,22]. We followed up on this by identifying another mutation in *STUB1* in the only family we have registered in our local database, presenting with a combination of ataxia and hypogonadotrophic hypogonadism, as well as additional symptoms possibly related to disease. As such, our data support the observation that mild to moderate and usually progressive cognitive impairment, is part of the clinical picture in *STUB1*-related ARCA. Importantly, despite their earlier onset of ataxia and more pronounced cognitive impairment, so far the patients in Family 1 have not experienced hormonal derangements as reported in some, but not all previously investigated families. This suggests that hypogonadotrophic hypogonadism may not be an obligatory feature of *STUB1*-related disease. We did not register pyramidal signs in our patients, in contrast to the observations of Synofzyk et al. [7]. However, they only found direct clinical evidence for pyramidal involvement in one family, and reported indirect pyramidal involvement in the other two, using central motor conduction time study. This was, however, not performed in our patients, due to the lack of clinical suspicion. Pyramidal signs were not reported in the other studies [5,8]. The main findings of our MRI analyses were severe cerebellar atrophy, as reported by previous studies with *STUB1* mutations, and in addition a distinct thinning of the anterior part of the corpus callosum (CC), not reported previously (Figure 2). Thin CC appears to be a common feature of many ARCAs [1]. Whether the thinning is a progressive feature, or whether it is associated with specific mutations, remain to be investigated. The combined data support mutations in *STUB1* as a rare cause of ARCA and broadens the clinical picture of the role of *STUB1* mutations in human disease.

Disorders of protein ubiquitination and thus protein turnover and homeostasis seem to be involved in both ataxias and neurodegenerative diseases. For neurodegenerative diseases such as Alzheimer and Parkinson, rare Mendelian forms have directly linked aberrations of the ubiquitination-proteasome system with the disease process [23-25]. In light of the range of mutations in various genes associated with ARCAs, the molecular mechanisms for similar disease are very complex and thus it is not surprising that the clinical picture is diverse. CHIP has at least two functions; as a co-chaperone for HSPs and other binding partners, and as an ubiquitin ligase [9,26]. In some ARCAs, part of the mechanism may involve the interaction between CHIP and ataxin-1 [11]. As CHIP has three domains with distinct functions, differences in patient phenotype could be related to the position of the mutation within the various domains. Our Family 1 is one of three reported with a mutation in the TPR domain. The patients reported by Synofzik et al. [7] also harboured mutations in the TPR domain, and the clinical report did not include hormonal derangements, thus it is possible that mutations affecting this domain do not predispose to hypogonadotrophic hypogonadism. Furthermore, although 3/4 of our patients had progressive disease, none of the patients showed progressive and debilitating dementia or white matter changes on MRI as reported in *RNF216* related ataxia [2]. In contrast to the findings of Synofzik et al. [7], none of our patients had spasticity. Very interestingly, features of accelerated ageing as observed in our Family 1, has not previously been reported in other *STUB*-related ARCA patients, but has been observed in CHIP knockout mice [15].

Our study documents at least two additional mechanisms whereby *STUB1* may contribute in the disease process. Firstly, we show that the *STUB1* mutations studied here result in a loss-of-function of CHIP, most likely related to decreased amount of CHIP protein. In patient fibroblasts, we see a drastic loss of available CHIP-E28K and CHIP-N65S protein compared to normal fibroblasts (CHIP-WT), while protein corresponding to the CHIP-K144* allele is completely absent. This is probably explained by the *in vivo* cell machinery detecting and marking most of the aberrant CHIP-proteins for degradation, since also low levels of mutant transcript was observed in our RNA analyses. We believe that reduced protein level, alone, is the mechanism for disease development in the compound heterozygous patient carrying the CHIP-K144* and the CHIP-E28K variant. Secondly, the causal mutation in Family 1 (CHIP-N65S) is located in the TPR domain of the protein, affecting a residue previously reported involved in substrate binding [21]. Based on the reduced ability of the CHIP-N65S mutant to ubiquitinate HSC70, reduced substrate affinity is thought to be a contributing factor to disease in this family, in addition to reduced protein level, as described above. Whether protein instability also contributes to the loss-of-function disease mechanism of the previously reported CHIP variants, is unknown. This possibility is supported by the phenotypic similarity reported between ARCA patients and the KO-mouse (CHIP^−^/^−^) model [15]. Mice lacking CHIP exhibit a deregulation of the protein quality control. Moreover, CHIP^−^/^−^ mice have a number of derangements including cardiomyopathy and accelerated aging. We did not find clinical evidence for cardiomyopathy in our patients, but all three siblings in Family 1 looked considerably older than their chronological age, and two had audiological findings compatible with slight presbyacusis while still young adults. Symptoms of accelerated ageing have not been reported in ARCAs before and could indicate difference in severity of the reported mutations, however these are only speculations. An age-related decrease in proteasome activity, but not specifically in CHIP activity, has been described [27]. The proteasome may be regarded as the downstream effector of the ubiquitin-proteasome system. Decreased activity may weaken cellular capacity to remove oxidatively modified proteins and thus promote the development of ageing. In addition, our index patient has diabetes type 1 and unexplained alopecia and his brother ulcerative colitis and uveitis, all of unknown etiology, but commonly regarded as autoimmune diseases. Whether or not this is due to chance or represents a causal link between *STUB1* mutation and dysregulation of ubiquitination in these diseases, remain to be seen. However, CHIP negatively modulates regulatory T cells in response to stress. CHIP cooperates with HSP70 [15,28] in ubiquitinating Foxp3, a central regulator of T cells, providing a glimpse of a theoretical mechanism for such a link.

## Conclusions

Taken together, our results demonstrate that *STUB1* mutations can cause ARCA by novel mechanisms such as protein instability and impaired substrate binding, leading to ataxia and hypogonadism. Additional features like accelerated ageing is possibly related to certain *STUB1* mutations, as seen in our Family 1, further refining the clinical spectrum of disease.

## Additional file

Additional file 1: Table S1. Variant filtration of exome sequencing data from the proband compared with whole genome genotyping data in all three affected siblings. Only one gene, *STUB1* harbors variants consistent with autosomal recessive inheritance and shared by all three siblings.
