# Supplementary material for: STUB1 mutations in autosomal recessive ataxias – evidence for mutation-specific clinical heterogeneity
Source: Orphanet J Rare Dis. 2014 Sep 26;9:146. doi: 10.1186/s13023-014-0146-0 (PMC4181732; doi:10.1186/s13023-014-0146-0)
Supplement: Additional file 1: Table S1. — Variant filtration of exome sequencing data from the proband compared with whole genome genotyping data in all three affected siblings. Only one gene, STUB1 harbors variants consistent with autosomal recessive inheritance and shared by all three siblings. [file 13023_2014_146_MOESM1_ESM.docx]

**Additional file 1: Table S1. Variant filtration of exome sequencing data from the proband compared with whole genome genotyping data in all three affected siblings.** Only one gene, *STUB1* harbors variants consistent with autosomal recessive inheritance and shared by all three siblings.

| **Filter** | **Count** |
| --- | --- |
| Exomic variants | 20438 |
| Excluding synonymous | 9872 |
| Not in 100 Norwegian exomes or in 1000Genomes (0.5% MAF) | 429 |
| Putative autosomal recessive genes | 32 |
| Shared by all three siblings | 1 |

**Table 1 – Clinical and radiological features of the four patients at examination date**

| Family-ID, Sex, Age at examination | II-1, male, 26 | II-2 male, 30 | II-3, female, 20 | II-1, female, 45 |
| --- | --- | --- | --- | --- |
| Substitution | N65S/N65S | N65S/N65S | N65S/N65S | E28K / K144* |
| Age of onset | 2 years | CP diagnosis at birth | 8 months | 33 years |
| Onset symptom | Delayed development | na | Delayed development | Oligomennorhea, secondary infertility |
| Dysmorphic features at examination | Aged appearance  Long slender fingers, increased space between digits four and five, adducted thumbs | Aged appearance  Adducted thumbs, | Aged appearance  Minor unspecific facial dysmorphism  Long slender fingers, increased space between digits four and five | None |
| First neurological symptom (age in years) | Gait impairment (17) | Gait impairment, dysarthria (12 ) | Gait impairment (15) | Gait ataxia , dysarthria (33) |
| Neurological signs & symptoms | Myokimies  Cerebellar ataxia (17 years), dysarthria  Dyspraxia  Increased muscle tone (rigidity)  Cognitive impairment | Head tremor and generalized intermittent postural tremor  Cerebellar ataxia, dysarthria, dysphagia  Increased muscle tone (rigidity and gegenhalten)  Distal muscle atrophy  Cognitive impairment | Dyspraxia  Decreased tempo  Cerebellar ataxia, mild dysarthria  Cognitive impairment  Epilepsy until 2 years of age | Cerebellar ataxia, Dysarthria, mild dysphagia  Retardation |
| Disability score* | 5 | 5 (from 22 years) | 2 | 4 |
| MR findings (at examination) | Cerebellar hypoplasia, thin posterior corpus callosum, mild thinning of pons | Severe cerebellar atrophy, thin CC, thin pons | Cerebellar hypoplasia, thin pons and corpus callosum | Cerebellar hypoplasia, mild thinning of pons, “empty sella” |
| Ophthalmological findings | Horizontal nystagmus | Left sided chronic iridocyclitis with secundary glaucoma; Oculomotor dyspraxia with saccadic pursuit | Horizontal nystagmus; mild retina atrophy | na |
| Endocrinology | Increased anti TPO  Diabetes type I |  | Delayed menarche for family | Secondary infertility Diabetes type X?  Hypothyroidism |
| Other | Alopecia  Slight presbyacusis | Ulcerative colitis | Slight presbyacusis | Pancreatitis |

- 0: no functional handicap; 2: no functional handicap but signs at examination; 2: mild, able to run, walking unlimited; 3: moderate, unable to run, limited walking without aid; 4: severe, walking with one stick; 5: walking with two sticks; 6: unable to walk, requiring wheelchair; 7: confined to bed.
